# S100A9-induced overexpression of PD-1/PD-L1 contributes to ineffective hematopoiesis in myelodysplastic syndromes

**DOI:** 10.1038/s41375-019-0397-9

**Published:** 2019-02-08

**Authors:** Pinyang Cheng, Erika A. Eksioglu, Xianghong Chen, Wendy Kandell, Thu Le Trinh, Ling Cen, Jin Qi, David A. Sallman, Yu Zhang, Nhan Tu, William A. Adams, Chunze Zhang, Jinhong Liu, John L. Cleveland, Alan F. List, Sheng Wei

**Affiliations:** 1 Department of Immunology, H. Lee Moffitt Cancer Center & Research Institute, Tampa, FL USA; 2 Cancer Biology PhD Program, University of South Florida and H. Lee Moffitt Cancer Center and Research Institute, Tampa, FL USA; 3 Bioinformatics Core, H. Lee Moffitt Cancer Center, Tampa, FL USA; 4 Department of Malignant Hematology, H. Lee Moffitt Cancer Center & Research Institute, Tampa, FL USA; 5 Department of Tumor Biology, H. Lee Moffitt Cancer Center & Research Institute, Tampa, FL USA

**Keywords:** Myelodysplastic syndrome, Anaemia

## Abstract

Myelodysplastic syndromes (MDS) are characterized by dysplastic and ineffective hematopoiesis that can result from aberrant expansion and activation of myeloid-derived suppressor cells (MDSCs) within the bone marrow (BM) niche. MDSCs produce S100A9, which mediates premature death of hematopoietic stem and progenitor cells (HSPCs). The PD-1/PD-L1 immune checkpoint impairs immune responses by inducing T-cell exhaustion and apoptosis, but its role in MDS is uncharacterized. Here we report an increased expression of PD-1 on HSPCs and PD-L1 on MDSCs in MDS versus healthy donors, and that this checkpoint is also activated in S100A9 transgenic (S100A9Tg) mice, and by treatment of BM mononuclear cells (BM-MNC) with S100A9. Further, MDS BM-MNC treated with recombinant PD-L1 underwent cell death, suggesting that the PD-1/PD-L1 interaction contributes to HSPC death in MDS. In accordance with this notion, PD-1/PD-L1 blockade restores effective hematopoiesis and improves colony-forming capacity in BM-MNC from MDS patients. Similar findings were observed in aged S100A9Tg mice. Finally, we demonstrate that c-Myc is required for S100A9-induced upregulation of PD-1/PD-L1, and that treatment of MDS HSPCs with anti-PD-1 antibody suppresses the expression of Myc target genes and increases the expression of hematopoietic pathway genes. We conclude anti-PD-1/anti-PD-L1 blocking strategies offer therapeutic promise in MDS in restoring effective hematopoiesis.

## Introduction

Myelodysplastic syndromes (MDS) are age-dependent hematopoietic stem cell malignancies characterized by dysplastic and ineffective hematopoiesis that result from abnormal and repressed bone marrow (BM) maturation [1–3]. Chronic inflammation coupled with senescence-dependent changes in both hematopoietic stem and progenitor cells (HSPCs) and the BM microenvironment are hallmarks of MDS pathogenesis [4–6]. In particular, excess generation of inflammatory cytokines, the expansion of regulatory T cells (Tregs), and upregulation or hyperactivation of immune receptors have been shown to drive the development of MDS [7–10]. Moreover, hematopoietic-inhibitory, myeloid-derived suppressor cells (MDSCs) are aberrantly expanded within the BM and are a paracrine source of S100A9, a proinflammatory protein and a damage-associated molecular pattern [9]. Notably, S100A9 induces expansion and activation of MDSCs, triggers cell death of HSPCs and myeloid and erythroid progenitors, and contributes to ineffective hematopoiesis [9, 10]. Finally, S100A9 transgenic mice (S100A9Tg) display age-dependent, excessive accumulation of MDSCs within the BM niche, and develop progressive cytopenias and multilineage cytological dysplasia. Thus, forced expression of S100A9 in vivo is sufficient to provoke hematopoietic alterations that phenocopy human MDS [9, 10].

The immune checkpoint receptor programmed cell death protein-1 (PD-1) and its ligand programmed cell death-ligand 1 (PD-L1) have emerged as important regulators of immune system homeostasis. PD-1 is expressed on the surface of activated T cells, whereas PD-L1 is predominantly expressed on antigen-presenting cells, but also on a variety of other immune cell types. PD-L1 expression is induced during inflammation and by tumor cells within the tumor microenvironment (TME) where it impairs the antitumor immune response [11]. Specifically, engagement of PD-L1 with PD-1 triggers a cascade of events that culminates in exhaustion/dysfunction of activated T cells [12, 13]. Germane to our studies, abnormal expression of PD-1 and PD-L1 has been implicated in MDS, where patients treated with azanucleosides and hypomethylating agents have shown an increased expression of checkpoint proteins post treatment [14–16]. Despite these findings, the strong association between inflammation and aging in MDS, and the regulation of the PD-1/PD-L1 axis, the role of these proteins in MDS pathobiology is unclear and unexplored.

Here we report a novel role for the PD-1/PD-L1 pathway in MDS beyond the regulation of T-cell biology, whereby aberrant activation of PD-1 on HSPCs by MDSC-derived PD-L1 triggers hematopoietic cell death, contributes to BM failure, and suppresses hematopoiesis. Most importantly, these data provide direct evidence that PD-1/PD-L1 blockade therapy, alone or in combination with other strategies, may have therapeutic potential to restore and promote effective hematopoiesis in MDS.

## Materials and methods

### MDS patients and human BM sample preparation

All patients were recruited from the Malignant Hematology clinic at the H. Lee Moffitt Cancer Center & Research Institute. After obtaining written informed consent, patients were classified according to World Health Organization and International Prognostic Scoring System criteria. The specimens used in this research were de-identified save for disease risk as shown in Supplementary Table S1. BM-mononuclear cells (MNC) were isolated from heparinized MDS BM and from heparinized BM of healthy human donors (purchased from Lonza-Walkersville). BM aspirates were first diluted twice their volume in 1× PBS. BM-MNC were then isolated by Ficoll-Hypaque gradient centrifugation as described [17]. After three washing steps, cells were maintained at 37 °C in 5% CO_2_-humidified atmosphere and cultured in RPMI-1640 medium supplemented with 10% FBS, 1% penicillin/streptomycin, and 1% l-glutamine (Gibco). For some experiments, fresh autologous plasma was used to stimulate BM-MNC (*n* = 5) at 20% v/v for 48 h.

### Animals

Wild-type FVB/NJ mice were purchased from Jackson Laboratory. S100A9Tg mice have been described [9]. Heterozygous c-Myc (*Myc*^+/−^) mice were derived from targeted embryonic stem cells [18, 19] and were backcrossed onto a pure C57BL/6J gene background for more than 15 generations. All mice were housed in pathogen-free units at the Division of Comparative Medicine vivarium at H. Lee Moffitt Cancer Center & Research Institute/University of South Florida. All animal experiments were approved by the Institutional Animal Care and Use Committee and performed in accordance with U.S. Public Health Service policy and National Research Council guidelines.

### Preparation of mouse BM cells

Each experimental cohort included 5–10 mice. Young and old mice were between 3–4 and 14–16 months old, respectively. Mouse BM cells were obtained from femurs and tibias, red blood cells were removed by ACK lysis buffer (Thermo Fisher Scientific), and BM cells were suspended in PBS with 2% bovine serum albumin (BSA).

### Mouse anti-PD-1 and anti-PD-L1 treatment

For in vivo anti-PD-1 blockade, the InVivoMAb anti-mouse PD-1 antibody was acquired from Bio X Cell. Nine-month-old S100A9Tg and age-matched wild-type FVB/NJ mice were administered 150 µg by intraperitoneal injection twice a week for 6 weeks; IgG2a was used as a control. Complete peripheral blood counts were performed using a ProCyte Dx Hematology Analyzer according to the manufacturer's protocol (IDEXX Laboratories). Mice were killed at the end of 6-week treatment, and BM cells were collected and used for flow cytometry analyses and colony formation assays.

### Flow cytometry

BM-MNC (1.5 × 10^6^ cells/sample) were stained with appropriate conjugated antibodies in PBS with 2% BSA. Specifically, human BM-MNC were stained with anti-CD33-PE-Cy7, anti-CD34-PerCP-Cy5.5, anti-CD14-BV510, anti-CD71-BV421, anti-CD38-BV711, anti-CD235a-BUV395, anti-PD-1-FITC, and anti-PD-L1-APC (BD Biosciences). Gating strategies for the identification of myeloid cells, HSPCs, and erythroid progenitors are shown in Supplementary Figure S1. Mouse BM cells were stained with anti-c-Kit-PerCP-Cy5.5, anti-Sca-1-PE, Lin-APC (including anti-CD3ε, anti-CD11b, anti-CD45R/B220, anti-TER-119, anti-Gr-1), anti-PD-1-BV421, anti-PD-L1-BV711, anti-PD-L2-BUV395, and anti-CD16/32-PE-Cy7 (BD Biosciences). Cell viability was determined using near-infrared live/dead dye (BD Biosciences) and the negative (live cell) population was used for further analysis. Samples were acquired on an LSR II flow cytometer and analyzed using FlowJo 9.9.3 software. Intracellular staining with PE-conjugated anti-active caspase-3 was performed using the Cytofix/Cytoperm™ protocol following initial cell surface receptor staining (BD Biosciences). For PD-1/PD-L1 ligation experiments, 2 million BM-MNC were plated per well in 24-well plates coated with recombinant human PD-L1 (2 μg/mL) for 24 h at 4 °C.

### Western blotting

Western blotting was performed as described previously [9]. Briefly, cell lysates were prepared by resuspending cell pellets in 1% NP-40, 10 mM Tris, 140 mM NaCl, 0.1 mM PMSF, 10 mM iodoacetamide, 50 mM NaF, 1 mM EDTA, 0.4 mM sodium orthovanadate, 10 μg/mL leupeptin, 10 μg/mL pepstatin, and 10 μg/mL aprotinin and lysing on ice for 30 min. Cell lysates were centrifuged at 12,000 × *g* for 15 min to remove nuclei and cell debris. Protein concentration of the soluble extracts was determined by using the Bradford protein assay (Bio-Rad). Fifty micrograms of protein (per lane) was separated by 10% SDS-PAGE and transferred to PVDF membranes, which were probed for indicated antibody: anti-PD-1 and anti-PD-L1 (Cell Signaling Technology); anti-c-Myc (Abcam); and anti-beta-actin (Sigma-Aldrich). Proteins were detected with ECL (GE Healthcare Amersham).

### Colony formation assay

Anti-mouse PD-1 and PD-L1, and anti-human PD-1 and PD-L1 Ultra-LEAF™ purified antibodies for neutralization were purchased from BioLegend. Two million BM-MNC were plated per well in 24-well plates and cultured with IgG (5 µg/mL), recombinant human S100A9 (rhS100A9; 5 µg/mL), anti-PD-1 (5 µg/mL), or anti-PD-L1 (5 µg/mL). After 48 h, cells were collected and used for colony formation assay. Healthy human donor or MDS patient BM-MNC were plated in duplicate in 35-mm culture dishes (1 × 10^5^ cells/dish) in complete methylcellulose media (Stemcell Technologies). Dishes were incubated at 37 °C in 5% CO_2_ for ~10–14 days, at which point burst-forming unit-erythroid (BFU-E) and colony-forming unit-granulocyte, monocyte (CFU-GM) colonies were counted using an inverted light microscope.

### RNA-seq and bioinformatics analysis

Total RNA from isolated CD34^+^ cells (isolated using StemExpress) from both healthy and MDS BM specimens was obtained using the RNeasy Mini Kit (Qiagen). RNA was quantified in a NanoDrop 1000 and RNA quality was assessed by Agilent 2100 Bioanalyzer. Samples were then processed for RNA-Sequencing (RNA-seq) using the NuGen Ovation Human RNA-Seq Multiplex System (NuGEN Technologies). Briefly, 100 ng of RNA was used to generate cDNA and a strand-specific library following the manufacturer’s protocol. Quality control steps included BioAnalyzer library assessment and quantitative PCR for library quantification. The libraries were then sequenced using an Illumina NextSeq 500 v2 sequencer with 75-base pair (bp)-end runs to generate ~60 million reads per sample. Sequencing reads were subjected to adaptor trimming, quality assessment, and were aligned to human reference genome hs37d5 using Tophat v2.0.13 [20]. Quantification of aligned sequences associated with each gene was performed using HTSeq v0.6.1 [21] based on GENCODE release 19. Read counts of all samples were normalized using the median-of-ratios method implemented in R/Bioconductor package DESeq2 v1.6.3. Differential expression analysis between PD-1 and IgG control-treated samples was performed by serial dispersion estimation and statistical model-fitting procedures implemented in DESeq2 [22]. Genes with a *p*-value, adjusted for multiple testing with the Benjamini–Hochberg correction, of less than 0.05 were determined to be significantly differentially expressed. Gene set enrichment analysis 3.0 software was used to assess significant enrichment of biological pathways or gene sets in PD-1 versus Ig-treated patient and healthy donor datasets, respectively, against hallmark gene sets from the Molecular Signatures Database v6.2 [23].

### Statistics

Data are presented as means ± standard error of the mean. Differences between individual groups were analyzed by Student’s *t*-test using Graphpad Prism version 7.03. *P* values < 0.05 were considered statistically significant. Significance was also confirmed with the Wilcoxon rank sum test.

## Results

### PD-1 and PD-L1 surface receptor expression is increased in MDS

**Fig. 1:**
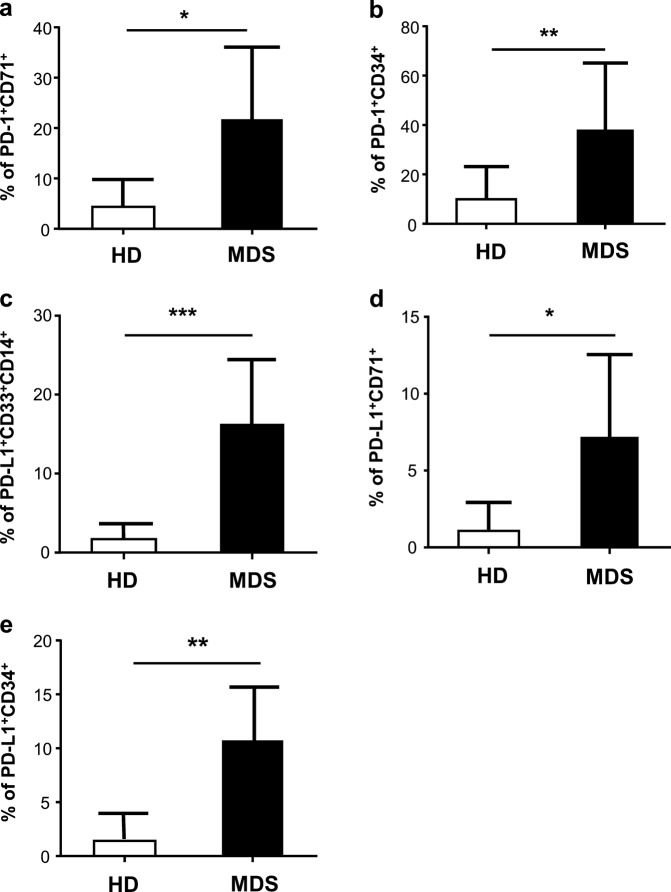
PD-1 and PD-L1 surface expression is increased in MDS. The percentage of PD-1^+^ and PD-L1^+^ hematopoietic cells was measured in BM-MNC isolated from MDS patients (*n* = 10) compared with healthy donors (*n* = 6): **a** PD-1^+^CD71^+^ erythroid progenitors; **b** PD-1^+^CD34^+^ HSPCs; **c** PD-L1^+^CD33^+^CD14^+^ MDSCs; **d** PD-L1^+^CD71^+^ erythroid progenitors; and **e** PD-L1^+^CD34^+^ HSPCs. Populations were gated on viable cells based on fluorescence minus one (FMO) controls (Supplementary Figure S2). *P < 0.05, **P < 0.01, ***P < 0.005; data are presented as mean ± standard error of the mean

Given the unexplored role of the PD-1/PD-L1 pathway in MDS, and the poorly understood function of PD-1 in non-lymphoid cells, we first examined PD-1 surface receptor expression on HSPCs and erythroid progenitors isolated from the BM of MDS patients (*n* = 10) compared with normal donors (*n* = 6) (Supplementary Figure S2). All patients examined had significantly increased surface expression of PD-1 on both CD71^+^ erythroid progenitors (*P* < 0.05; Fig. 1a) and CD34^+^ HSPCs (*P* < 0.01; Fig. 1b) versus the corresponding healthy donor BM populations, suggesting PD-1 upregulation may play a role in MDS. As PD-L1 ligation with PD-1 mediates immune suppression in the TME [24], we examined PD-L1 expression on CD33^+^CD14^+^ myeloid cells in BM, which represent MDSCs. CD33^+^CD14^+^ MDSCs from MDS BM showed marked upregulation of PD-L1 surface expression versus that on MDSC from the BM of normal donors (*P* < 0.005; Fig. 1c). Finally, erythroid progenitors (*P* < 0.05; Fig. 1d) and HSPCs (*P* < 0.01, Fig. 1e) from MDS patients also demonstrated significantly increased surface expression of PD-L1 versus expression in normal progenitors, indicating an autonomous regulation of immune checkpoint signaling in these MDS hematopoietic populations.

**Fig. 2:**
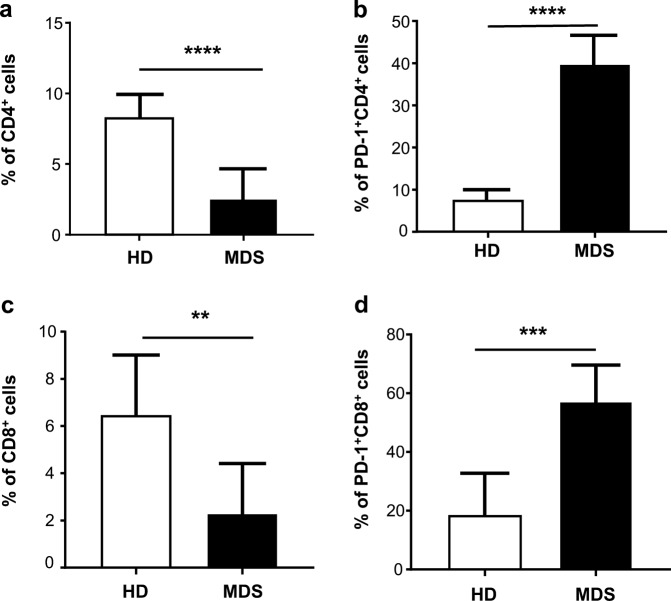
PD-1 expression is increased in MDS patient T cells present in the bone marrow niche. The percentage of CD4^+^ and CD8^+^ T cells and their expression of PD-1 were measured in BM-MNC isolated from MDS patients (*n* = 10) compared with healthy donors (*n* = 6): **a** percent CD4^+^ T cells; **b** percent CD4^+^PD-1^+^ cells; **c** percent CD8^+^ T cells; and **d** percent CD8^+^PD-1^+^ cells. Populations were gated on viable cells based on fluorescence minus one (FMO) controls. **P < 0.01, ***P < 0.005, ****P < 0.001; data are presented as mean ± standard error of the mean

As PD-1/PD-L1 changes are most prominently studied in T cells in other tumor types, we also studied this population in MDS specimens. Total T cells, including both CD4^+^ and CD8^+^ T cells, were significantly reduced in primary MDS BM-MNC (Fig. 2a, c, and Supplementary Figure 3a; *P* < 0.001, *P* < 0.01, and *P* < 0.01, respectively), and the PD-1 expression was increased in these cells versus CD4^+^ and CD8^+^ T cells from normal BM-MNC (Fig. 2b, d; *P* < 0.001 and *P* < 0.005, respectively). Finally, there was an upregulation of Tregs in MDS BM-MNC compared to healthy controls (Supplementary Figure 3b, *P* < 0.01). We conclude that the MDS BM niche is immune suppressive.

### PD-1 and PD-L1 are overexpressed in aged S100A9Tg mice

**Fig. 3:**
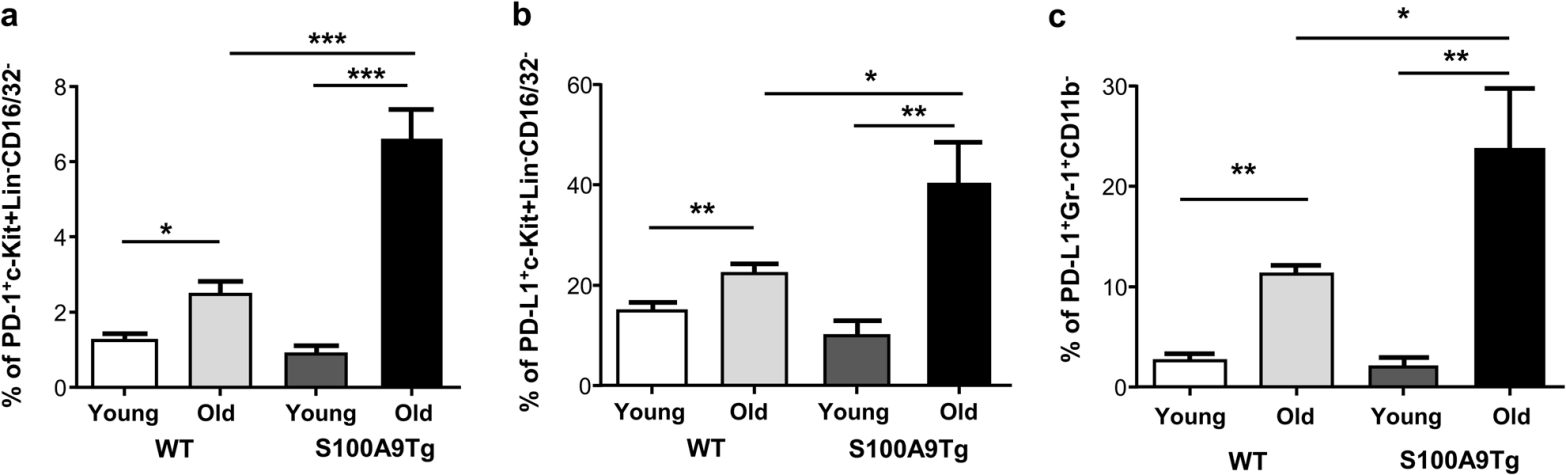
PD-1 and PD-L1 expression levels are elevated in the CMP and MDSC of S100A9Tg mice. The percentage of PD-1^+^ and PD-L1^+^ hematopoietic cells was measured in BM isolated from age-matched (young, 3–4 months; old, 14–16 months) S100A9Tg and WT littermates. Five mice were used per group and the experiment was repeated three times. **a** PD-1^+^c-Kit^+^Lin^−^CD16/32^−^ CMP cells; **b** PD-L1^+^ CMP cells; and **c** Gr-1^+^CD11b^-^ MDSCs. Populations were gated on viable cells based on fluorescence minus one (FMO) controls (Supplementary Figure S3). *P < 0.05, **P < 0.01, ***P < 0.005; data are presented as mean ± standard error of the mean

Akin to human MDS, S100A9Tg mice, where S100A9 expression is driven by the H2K (myeloid) promoter, display age-dependent activation of MDSC that results in hematopoietic progenitor cell death and dysplastic, ineffective hematopoiesis [9]. Using this mouse model, we assessed the expression of PD-1 and PD-L1 in the BM-MNC and MDSC of young and old S100A9Tg mice (3–4 and 14–16 months old, respectively) compared with age-matched wild-type (WT) littermates (Supplementary Figure S4). c-Kit^+^Lin^−^CD16/^−^CD32^−^ common myeloid progenitor (CMP) cells derived from BM of young WT and S100A9Tg mice showed little expression of PD-1 and PD-L1 expression on (Fig. 3a, b). In contrast, PD-1 and PD-L1 expression levels on CMPs were significantly greater in older versus younger mice, and there were marked increases in PD-1 (Fig. 3a, *P* < 0.005) and PD-L1 (Fig. 3b, *P* < 0.05) expression in aged S100A9Tg mice versus aged WT littermates; thus, S100A9 induces PD-1 and PD-L1 surface expression beyond that which is manifest with normal aging. Notably, increased PD-L1 surface receptor expression was also observed on Gr-1^+^CD11b^-^ cells that represent MDSCs in S100A9Tg mice (Fig. 3c). Thus, forced expression of S100A9 is sufficient to augment an immune suppression PD-L1/PD-1 circuit driven by MDSC.

Given T cell deficits in the BM niche of MDS patients, we also assessed T-cell numbers in S100A9Tg mice. There was no statistically significant difference in the numbers of CD4^+^ or CD8^+^ cells in WT versus S100A9Tg littermates, although increased PD-1 expression was observed in the CD8^+^ cells of S100A9Tg mice versus WT littermates (Supplementary Figure 4d). Comparing the difference in T-cell PD-1 expression between human and mice suggests the PD-1/PD-L1 axis changes in T cells may be independent of its effects on HSPC and hence their function may be more in immunesurveillance rather than hematopoietic suppression.

### S100A9 directly induces PD-1 expression on progenitors and PD-L1 on MDSC

**Fig. 4:**
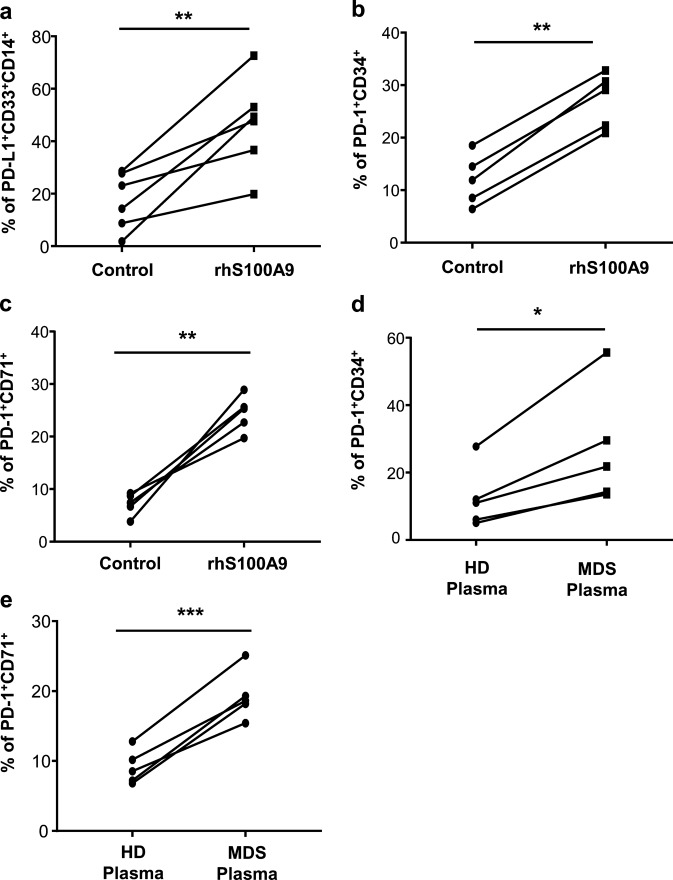
S100A9 induces the expression of PD-1 on progenitors and PD-L1 on MDSCs. The percentage of PD-1^+^ and PD-L1^+^ hematopoietic cells was measured in BM-MNC isolated from healthy donors (*n* = 5) treated with 10 μg/mL rhS100A9 or control IgG (control) (**a-c**), or with MDS patient or healthy donor BM plasma (**d, e**) for 48 h. **a** PD-L1^+^CD33^+^CD14^+^ MDSC; **b** PD-1^+^CD34^+^ HSPC; **c** PD-1^+^CD71^+^ erythroid progenitors; **d** PD-1^+^CD34^+^ HSPCs; and **e** PD-1^+^CD71^+^ erythroid progenitors. Populations were gated on viable cells based on fluorescence minus one (FMO) controls (Supplementary Figure S4). *P < 0.05, **P < 0.01; data are presented as mean ± standard error of the mean

To test if S100A9 can directly induce PD-1 and PD-L1 cell surface expression in primary hematopoietic cells, BM-MNC isolated from healthy donors (*n* = 5) were treated with 10 μg/mL recombinant human S100A9 (rhS100A9) or IgG (control) for 48 h (Supplementary Figure S5). Treatment with rhS100A9 led to significant increases in PD-L1 expression on CD33^+^CD14^+^ MDSCs in all healthy donors analyzed (Fig. 4a; *P* < 0.01). Furthermore, rhS100A9 treatment also led to significant increases in PD-1 cell surface expression on both CD34^+^ HSPCs (Fig. 4b; *P* < 0.01) and CD71^+^ erythroid progenitors (Fig. 4c; *P* < 0.01).

BM plasma concentrations of S100A9 are significantly elevated in MDS patients compared with healthy donors [9, 10]. To test if BM plasma-derived S100A9 can induce surface receptor expression of PD-1 and PD-L1, healthy donor BM-MNC were incubated with each MDS patient plasma (patients 1, 3, 7, 12, and 15 from Supplementary Table 1). In accordance with the effects of rhS100A9 treatment, incubation of healthy donor BM-MNC with MDS patient plasma triggered significant increases in the percentage of PD-1^+^CD34^+^ HSPCs (Fig. 4d; *P* < 0.05) and PD-1^+^CD71^+^ erythroid progenitors (Fig. 4e; *P* < 0.005). Thus, S100A9 directly induces the expression of PD-1 on HSPCs and its corresponding ligand PD-L1 on MDSC.

### The S100A9 to PD-1/PD-L1 axis induces hematopoietic cell death

**Fig. 5:**
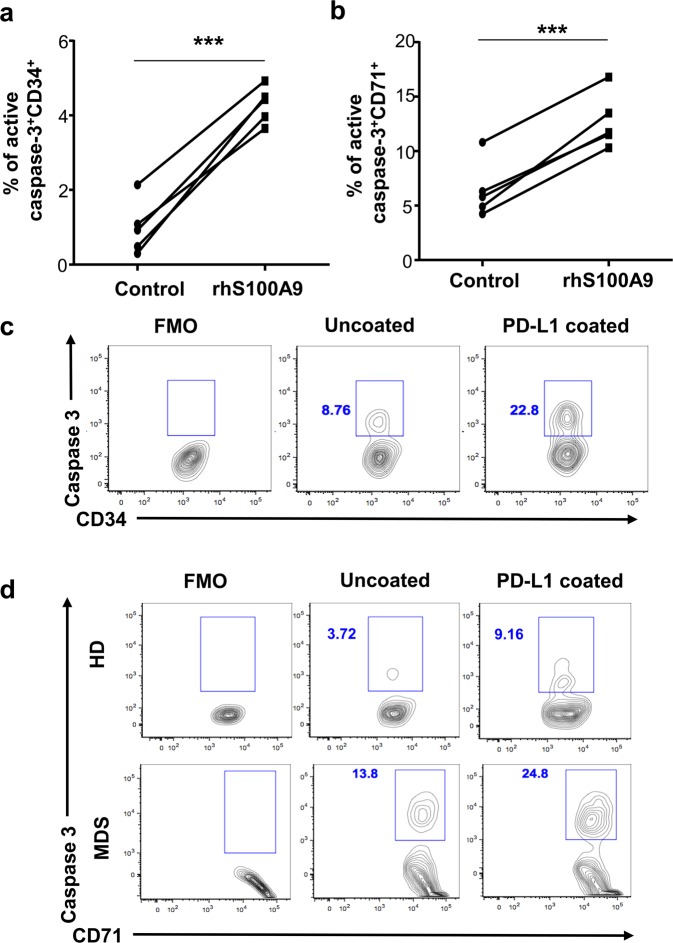
S100A9/PD-1/PD-L1 axis induces hematopoietic cell death. **a, b** The percentage of active caspase-3^+^ hematopoietic cells was measured in BM-MNC isolated from healthy donors (*n* = 5) treated with 10 μg/mL rhS100A9 or control IgG (control) for 48 h. Populations were gated on viable cells based on fluorescence minus one (FMO) controls: percentage of active caspase-3 **a** CD34+ HSPCs and **b** CD71+ erythroid progenitors. ***P < 0.001; data are presented as mean ± standard error of the mean. **c, d** Two million BM-MNC were plated per well in 24-well plates coated with recombinant human PD-L1 (2 μg/mL) for 24 h at 4 °C. The percentage of active caspase-3^+^**c** CD34^+^ HSPCs and **d** CD71^+^ erythroid progenitors are shown in a representative sample from three healthy donors and MDS patients

PD-1 and PD-L1 engagement induces T-cell exhaustion and apoptosis [12, 13]. Thus, we tested if PD-1 engagement on HSPCs with PD-L1 induces cell death and contributes to the ineffective hematopoiesis that is a hallmark of MDS. BM-MNC isolated from healthy donors (*n* = 5) were treated with 10 μg/mL rhS100A9 or IgG (control) for 48 h, and cells were then assessed for levels of surface PD-1 and PD-L1 and anti-active caspase-3 expression, a marker of cell death. The percentage of CD34^+^ HSPCs and CD71^+^ erythroid progenitors with active caspase-3 was significantly increased by rhS100A9 treatment (Fig. 5a, b; *P* < 0.005). To assess if engagement of PD-1 was also sufficient to provoke hematopoietic progenitor cell death and if this was augmented in MDS, BM-MNC isolated from MDS patients (*n* = 3) and healthy donors (*n* = 3) were cultured in plates coated with or without recombinant human PD-L1 for 24 h and levels of activated caspase-3 were determined by flow cytometry. PD-1/PD-L1 ligation resulted in marked increases in activated caspase-3 in CD34^+^ HSPCs and CD71^+^ erythroid progenitors from MDS patients but only slightly in normal donors (Fig. 5c, d). Thus, PD-1/PD-L1 interactions may contribute to progenitor cell death that is manifest in MDS.

### PD-1/PD-L1 pathway blockade promotes effective hematopoiesis in MDS

**Fig. 6:**
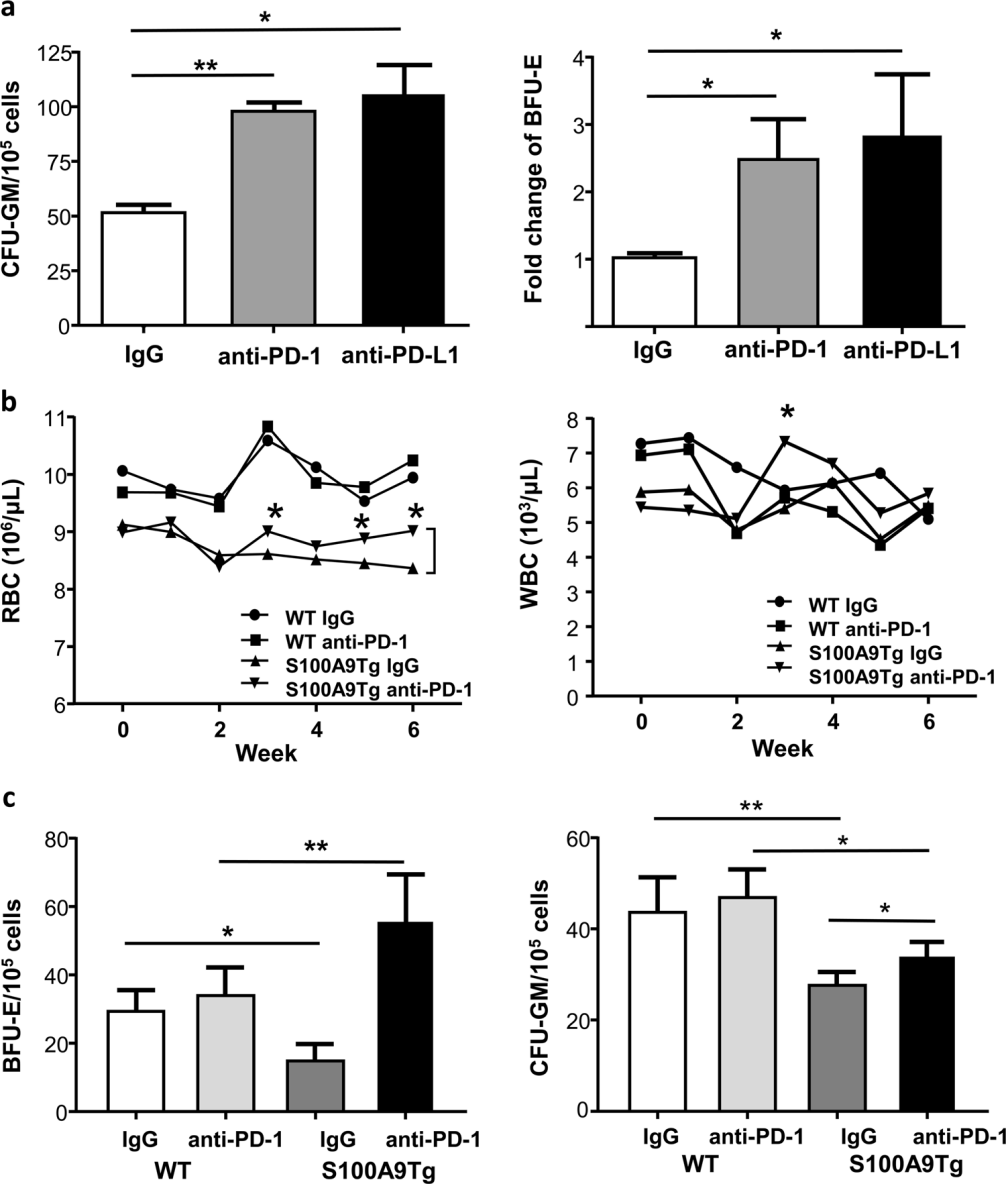
PD-1/PD-L1 pathway blockade augments the colony-forming capacity of MDS patient BM progenitors and improves hematopoiesis in S100A9Tg mice. **a** MDS BM-MNC (*n* = 5) were treated with anti-PD-1 or anti-PD-L1 blocking antibody for 48 h, and cells were then plated to assess CFU-GM and BFU-E colony-forming capacity versus cells treated with control IgG. **b, c** Aged (14 months) S100A9Tg (*n* = 6) and age-matched WT mice (*n* = 6) were treated with 150 μg/mouse anti-PD-1 blocking antibody twice a week for 6 weeks. **b** Complete blood counts were measured weekly, and mean RBC and WBC counts are shown through week 6. **c** BM cells from the aged, anti-PD-1-treated S100A9Tg and WT mice were isolated and BFU-E and CFU-GM colony-forming capacity was determined. *P < 0.05, **P < 0.01; data are presented as mean ± standard error of the mean. BFU-E burst forming unit-erythroid; CFU-GM colony-forming unit-granulocyte, monocyte; RBC red blood cell; WBC white blood cell

To test if blocking PD-1/PD-L1 interaction could improve hematopoiesis in MDS, colony-forming capacity was assessed after treating MDS BM-MNC (*n* = 5) with anti-PD-1 or anti-PD-L1 blocking antibody for 48 h. Notably, PD-1 and PD-L1 blockade significantly improved CFU-GM (*P* < 0.01 and *P* < 0.05, respectively) and BFU-E (*P* < 0.05 for both) colony-forming capacity versus IgG control-treated BM-MNC (Fig. 6a), suggesting PD-1/PD-L1 blockade may be a beneficial treatment option to promote effective hematopoiesis in MDS. To assess if hematopoietic improvement with PD-1 blockade is also manifest in vivo, aged S100A9Tg (*n* = 6) and WT littermates (*n* = 6) were treated with 150 μg/mouse anti-PD-1 blocking antibody twice a week for 6 weeks, and complete blood counts were measured weekly. Anti-PD-1 treatment of S100A9Tg mice significantly increased red blood cell (RBC) and white blood cell (WBC) counts versus IgG controls (Fig. 6b). In contrast, anti-PD-1 treatment of WT mice had no effect on RBC and WBC counts, as expected given that WT BM progenitors express low levels of PD-1 and PD-L1 (Fig. 3).

To test if increased complete blood counts in S100A9Tg mice reflected improved hematopoiesis, BM cells from the anti-PD-1-treated S100A9Tg and WT mice were isolated to assess colony-forming capacity. As expected, BFU-E and CFU-GM colony-forming capacity was significantly reduced in S100A9Tg versus WT mice treated with control IgG (*P* < 0.05; Fig. 6c). Importantly, anti-PD-1-treated S100A9Tg mice had significant increases in BFU-E colony-forming capacity versus WT-treated mice (Fig. 6c, *P* *<* 0.01). There were also modest increases of CFU-GM in anti-PD-1-treated S100A9Tg mice. Similarly, BFU-E and CFU-GM in S100A9Tg BM were significantly increased with anti-PD-L1 treatment (Supplementary Figure S6). These data suggest that PD-1 and PD-L1 blockade is an attractive therapeutic strategy to restore effective hematopoiesis in MDS patients.

### S100A9-induced expression of PD-1 and PD-L1 is associated with abnormal c-Myc activation

**Fig. 7:**
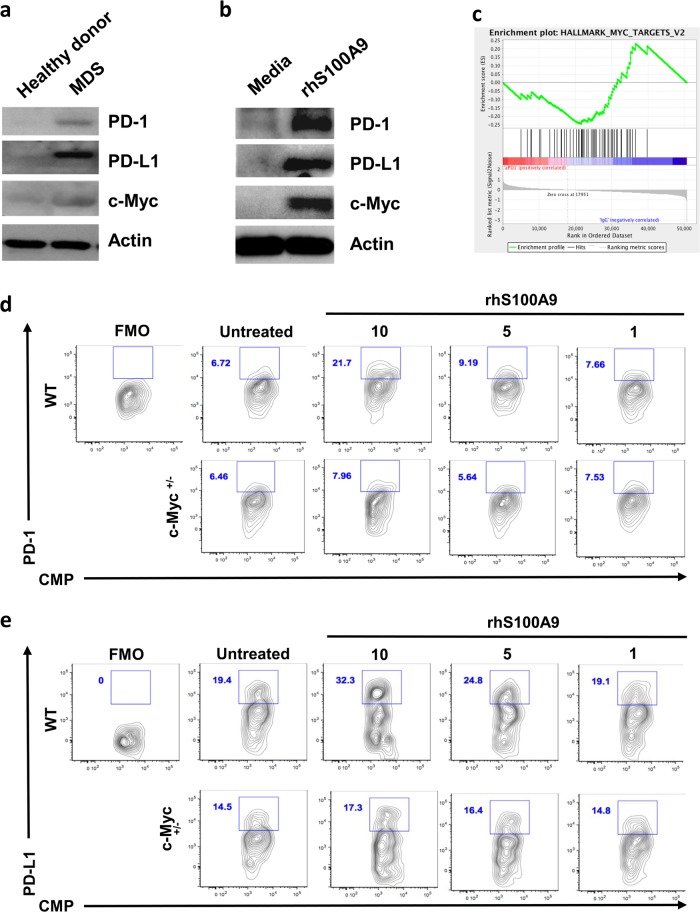
S100A9/PD-1/PD-L1 pathway activation augments MYC expression. Western blot was performed to assess levels of PD-1, PD-L1, and MYC protein in **a** MDS versus healthy donor BM-MNC and **b** healthy donor BM-MNC treated with 10 μg/mL rhS100A9 for 48 h. Data are representative of five independent experiments. (**c**) RNA-seq-based transcriptional network analysis indicating downregulation of Hallmark Myc pathways in anti-PD1-treated CD34+ HSPC (*n* = 5). The percentage of PD-1^+^ and PD-L1^+^ c-Kit^+^Lin^−^CD16/32^−^ CMP cells was measured in BM cells isolated from *Myc*^+/−^ and WT littermates and treated with increasing concentrations of S100A9. Populations were gated on viable cells based on fluorescence minus one (FMO) controls. The percentage of **d** PD-1^+^ and **e** PD-L1^+^ CMP cells in the BM of representative samples from five *Myc*^+/−^ and WT mice is shown

Recent studies have suggested that abnormal metabolic changes driven by the c-Myc (MYC) oncogenic transcription factor during tumorigenesis lead to PD-1/PD-L1 pathway activation and immunosuppression [11, 25]. Aged S100A9Tg mice display robust activation of S100A9-induced inflammatory pathways that lead to abnormal metabolic changes, as evidenced by increased body weight, insulin resistance, and hyperglycemia [26]. To assess the possible links of S100A9/PD-1/PD-L1 pathway activation and MYC in MDS, MYC protein levels were compared in BM-MNC from MDS patients versus healthy donors. Consistently (*n* = 5 total), PD-1, PD-L1, and MYC protein expression were elevated in MDS BM-MNC versus healthy donors (Fig. 7a). Further, the treatment of healthy donor BM-MNC with 10 μg/mL rhS100A9 for 48 h was sufficient to induce the expression of PD-1, PD-L1, and MYC versus untreated cells (Fig. 7b). Conversely, RNA-seq analyses of MDS HSPC treated or not treated with anti-PD-1 for 48 h revealed a decrease in MYC target genes following treatment with anti-PD-1, along with increases in the expression of genes involved in heme metabolism and IL2/STAT5 signaling, as would be expected with improvements in hematopoiesis (Fig. 7c, and Supplementary Figure S7a).

To test if Myc contributes to S100A9-directed increases in the cell surface expression of PD-1 and PD-L1 on BM-MNC, we assessed the effects of Myc heterozygosity using BM from heterozygous *Myc*^+/−^ mice and WT littermates; *Myc*^+/−^ BM cells expressed reduced levels of Myc protein versus WT littermate BM cells as judged by immunoblots (Supplementary Figure S7b). These cells were then treated with increasing concentrations of S100A9 for 48 h and PD-1 and PD-L1 cell surface expression was assessed on c-Kit^+^Lin^−^CD16/32^−^ CMP cells by flow cytometry. The surface receptor expression of PD-1 (Fig. 7d top panel) and PD-L1 (Fig. 7e top panel) increased in an S100A9 concentration-dependent manner on WT-derived BM cells. Conversely, S100A9 treatment was insufficient to induce PD-1 and PD-L1 cell surface receptor expression in heterozygous *Myc*^+/−^ BM cells (Fig. 7d, e bottom panels). Thus, Myc contributes to the control of the PD-1/PD-L1 axis by S100A9.

**Fig. 8:**
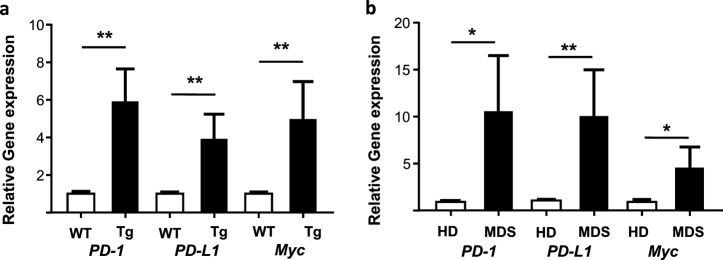
*MYC*, *PD-1,* and *PD-L1* expression levels are elevated in the BM-MNC of MDS patients and of S100A9Tg mice. **a** qRT-PCR analysis of BM-MNC isolated from WT (*n* = 5) and S100A9Tg (*n* = 5) aged littermate mice were analyzed for the expression of *PD-1*, *PD-L1*, and *Myc*. **b** qRT-PCR analysis of BM-MNC isolated from healthy (*n* = 5) versus MDS (*n* = 5) patients. *P < 0.05, **P < 0.01, ***P < 0.005; data are presented as mean ± standard error of the mean

To gain insights into the mechanism by which S100A9 controls the expression of PD-1, PD-L1, and Myc, quantitative real-time PCR analyses were performed. These studies revealed that *PD-1*, *PD-L1*, and *Myc* mRNA levels are elevated in S100A9Tg versus WT BM-MNC (Fig. 8a) and in MDS patient versus normal BM-MNC (Fig. 8b). Collectively these findings suggest that S100A9 induction of Myc triggers increases in PD-1 and PD-L1 expression that activate MDSC, provoke HSPC cell death, and lead to immune evasion.

## Discussion

Despite recent advances in our understanding of MDS pathobiology, reversing ineffective hematopoiesis remains a major therapeutic challenge, given that the precise signaling pathways mediating HSPC defects are not fully understood. Our recent investigations have shown that a chronic inflammatory response, coupled with expansion of hematopoietic-inhibitory MDSCs, directs HSPC injury and clonal selection in MDS [9, 10, 27]. Within the BM niche, MDSCs serve as a paracrine source of the alarmin S100A9, which activates and expands MDSCs, and is sufficient to provoke cell death of HSPCs to drive ineffective hematopoiesis [9, 10]. These findings have been validated by others in alternate models, namely deletion 5q (del[5q]) MDS and the *Rps14*-haplodeficient murine model, whereby S100A9 and S100A8 (calprotectin), the heterodimeric binding partner of S100A9, trigger erythroid progenitor cell death [28].

An emerging concept is that the local BM microenvironment in MDS is also abnormal and plays critical roles in MDS pathobiology. Here, aging-associated inflammation, or “inflammaging,” likely contributes to genetic instability and a suppressive BM microenvironment, and to defective HSPCs [9, 10, 29–35]. Indeed, S100A9 expression is also induced in mesenchymal stromal cells, where it contributes to DNA damage in HSPCs via reactive oxygen species and p53-dependent pathways [36]. Importantly, S100A9 is also implicated in clonal expansion and leukemia progression in both primary MDS specimens and in the S100A9Tg mouse model that phenocopies human MDS [36]. Finally, S100A9 can also suppress the transcription and cellular elaboration of erythropoietin [37]. Collectively, these findings establish central roles for S100A9 in MDS pathobiology, whereby aberrant S100A9-driven signaling results in pathobiological consequences in the entire BM niche.

To prevent unwarranted damage and maintain homeostasis, normal proinflammatory responses include events that suppress inflammation and dampen the immune response. One such mechanism of the immune system is governed by the immune checkpoint receptor PD-1 and its ligand PD-L1, where engagement of PD-1 induces T-cell exhaustion and apoptosis [12, 13]. Strikingly, here our studies revealed nontraditional roles (i.e., non-T-cell-specific) for PD-1 and PD-L1 in MDS, where S100A9 contributes to ineffective hematopoiesis in MDS via induction of PD-1 and PD-L1 expression. Importantly, checkpoint inhibitor treatment both in vitro and in vivo is shown to improve hematopoiesis in MDS, supporting the notion that anti-PD-1 or anti-PD-L1 alone or in combination with other therapies will show a benefit for MDS patients.

MDS patients express increased levels of PD-1 and PD-L1 on CD34^+^ HSPCs and CD71^+^ erythroid progenitors versus age-matched healthy donors, whereas CD33^+^CD14^+^ MDSC express elevated levels of PD-L1 in MDS. Similar increases in PD-1 and PD-L1 expression are manifest in aged S100A9Tg mice and in healthy donor BM-MNC treated with rhS100A9; thus, S100A9 is sufficient to provoke increases in PD-1 and PD-L1 expression. Further, PD-L1 can directly induce CD34^+^ HSPC and CD71^+^ erythroid progenitor cell death, suggesting PD-1/PD-L1 ligation contributes to ineffective hematopoiesis in MDS. Consistent with this notion, PD-1 or PD-L1 blockade markedly improves the hematopoietic colony-forming capacity of MDS BM-MNC. Similarly, in vivo treatment of S100A9Tg mice with PD-1 or PD-L1 blocking antibody significantly improves hematopoiesis, as evidenced by improved complete blood cell counts and increased colony-forming capacity. Collectively, these data highlight the therapeutic potential of PD-1/PD-L1 pathway blockade in MDS.

Notably, Myc is known to induce the transcription of PD-L1 [11], and our studies have shown that Myc contributes to S100A9-mediated control of PD-1/PD-L1 surface receptor expression. Interestingly, Myc is a major regulator of tumor cell metabolism, and here we have found that BM-MNC of MDS patients and healthy BM-MNC treated with rhS100A9 express elevated Myc levels, and that MDS patients and S100A9Tg mice display features of metabolic syndrome [38], including hyperglycemia in the BM niche. We posit that high concentrations of S100A9 produced by MDSCs in the BM niche, as well as other inflammatory cytokines such as IL-10 and TGF-β, provoke an abnormal metabolic profile via activation of Myc. While the mechanisms of Myc activation downstream of S100A9 will need to be elucidated, our data have previously demonstrated the activation of β-catenin which has as its downstream targets Myc [10, 39]. Regardless of the precise wiring, S100A9 control of PD-1/PD-L1 is shown to clearly involve Myc, which is known to induce *PD-L1* transcription in tumors to facilitate immune evasion [25], as *Myc* heterozygosity significantly dampens the expression of these checkpoints.

S100A9-directed control of the PD-1/PD-L1 axis also has clinical implications. First, S100A9 expression appears unaffected by the epigenetic drug 5-azacytidine [40], and S100A9-directed induction of PD-1/PD-L1 may contribute to therapeutic resistance of MDS to 5-azacytidine, which also induces the expression of these immune checkpoints [15]. Second, our RNA-seq analysis of primary human MDS HSPC treated with anti-PD-1 antibody suggests the relevance of the S100A9-PD-1/PD-L1 circuit would also affect the use of erythropoietin as a hematopoietic stimulating agent in combination with anti-PD-1/PD-L1 treatment. Specifically, anti-PD-1 treatment induces the expression of the heme metabolic pathway genes as well as the Epo/STAT5 pathway, which are known regulators of erythroid cell proliferation and survival in MDS [41, 42]. This suggests that the improved BFU-E colony formation of MDS BM after treatment with anti-PD-1/PD-L1 in our studies involves Epo/STAT5 regulation, which will be an important aspect of future analyses.

Collectively, these findings (1) expand our understanding of the role of PD-1/PD-L1 interaction beyond T cells, which has implications for general immunology and immunotherapy; (2) demonstrate that S100A9, which is instrumental in the pathogenesis of MDS, plays a critical role in the induction of PD-1/PD-L1 surface receptor expression on HSPCs and MDSCs; and (3) suggest that PD-1/PD-L1 signaling contributes to ineffective hematopoiesis in MDS. Finally, and importantly, these data suggest that anti-PD-1 or anti-PD-L1 blocking antibodies, alone or in combination with other strategies, offer therapeutic promise in MDS to improve the BM microenvironment and restore effective hematopoiesis.

## Supplementary information

Supplemental figures and legends
