# Supplementary material for: S100A9-induced overexpression of PD-1/PD-L1 contributes to ineffective hematopoiesis in myelodysplastic syndromes
Source: Leukemia. 2019 Feb 8;33(8):2034–46. doi: 10.1038/s41375-019-0397-9 (PMC6687540; doi:10.1038/s41375-019-0397-9)
Supplement: Supplementary file 1 — Supplemental figures and legends [file 41375_2019_397_MOESM1_ESM.docx]

**Supplementary Material**

S100A9-induced expression of PD-1/PD-L1 contributes to ineffective hematopoiesis in myelodysplastic syndromes

**Pinyang Cheng^1^, Erika A. Eksioglu^1^, Xianghong Chen^1^, Wendy Kandell^2^, Thu Le Trinh^1^, Jin Qi^1^, David A. Sallman^3^, Yu Zhang^1^, Nhan Tu^1^, William A. Adams^1^, Jinhong Liu^1^, John L. Cleveland^4^, Alan F. List^3^ and Sheng Wei^1^**

^1^Department of Immunology, H. Lee Moffitt Cancer Center & Research Institute, Tampa, FL, USA; ^2^Cancer Biology PhD Program, University of South Florida and H. Lee Moffitt Cancer Center and Research Institute, Tampa, FL, USA; ^3^Department of Malignant Hematology, H. Lee Moffitt Cancer Center & Research Institute, Tampa, FL, USA; ^4^Department of Tumor Biology, H. Lee Moffitt Cancer Center & Research Institute, Tampa, FL, USA.

Supplementary materials include:

Supplementary Figure Legends

Supplementary Figures S1-S7

Supplementary Table S1

**SUPPLEMENTARY FIGURE LEGENDS**

**Supplementary Figure S1.** **Flow cytometry gating strategies for human BM-MNCs.** Human BM-MNCs were stained with antibodies: anti-CD33-PE-Cy7, anti-CD34-Percp-Cy5.5, anti-CD14-BV510, anti-CD71-BV421, anti-CD38-BV711, anti-CD235a-BUV395, anti-PD-1-FITC and anti-PD-L1-APC. Near infrared live/dead dye was used for distinction of live or dead cells. Flow acquisitions were performed using LSR II cytometer and analysis was done using live cells with flowjo.

**Supplementary Figure S2.** **PD-1 and PD-L1 gating strategy on representative human BM-MNCs. a)** PD-1 expression on erythroid precursors CD14^-^CD33^-^CD34^-^CD71^+^CD235a^+^ cells: cells positive for PD-1 surface expression were calculated based on FMO gate setting. **b)** PD-1 expression on progenitors: Positive PD-1 surface expression on CD14^-^CD33^-^CD71^-^CD34^+^CD38^+^ progenitors was determined using FMO gates. **c)** PD-L1 expression on CD14^+^CD33^+^ cells. **d)** PD-L1 expression on erythroid precursors CD14^-^CD33^-^CD34^-^CD71^+^CD235a^+^ cells. **e)** PD-L1 expression on CD14^-^CD33^-^CD71^-^CD34^+^CD38^+^ progenitors.

**Supplementary Figure S3.** **Reduced total T cell population and increased T reg in MDS. a)** Human BM-MNCs were stained with antibodies: anti-CD3-V450, anti-CD4-BUV395, anti-CD8-Percp-Cy5.5, anti-PD-1-PE, and anti-PD-L1-APC. Near infrared live/dead dye was used for distinction of live or dead cells. Percentage of CD3^+^ T cells in whole population was calculated based on live cells. **b)**  Human BM-MNCs were stained with antibodies: anti-CD4-BUV395, anti-CD8-Percp-Cy5.5, anti-CD25-BV421, antiCD33-BV786, and anti-CD14-BV510 for cell surface staining. After fixation/permeabilization, cells were stained with anti-Foxp3-PE. Treg were gated CD33^-^CD14^-^CD4^+^CD25^+^Foxp3^+^ cells. Percentage of Treg cells in whole population was calculated.

**Supplementary Figure S4.** **PD-1 and PD-L1 gating strategy on representative murine BM cells.** FVB/NJ mice bone marrow cells were stained with Abs: anti-c-Kit-Percp-Cy5.5, anti-Sca-1-PE, Lin-APC (including anti-CD3e, anti-CD11b, anti-CD45R/B220, anti-TER-119, anti-Gr-1), anti-PD-1-BV421, anti-PD-L1-BV711, anti-PD-L2-BUV395, and anti-CD16/32-PE-Cy7. Near infrared live/dead dye was used for distinction of live or dead cells. For MDSC staining, antibodies used for staining were: anti-CD11b-PE-Cy7, anti-Gr-1-Percp-Cy5.5, anti-PD-1-BV421, anti-PD-L1-BV711, anti-PD-L2-BUV395. For T cell staining, antibodies used were: anti-CD3-PE, anti-CD4-BV510, anti-CD8-BUV395, anti-PD-1-BV421, anti-PD-L1-BV711. Flow acquisitions were performed using LSR II cytometer and analysis was done using live cells with FlowJo. **a)** PD-1 expression on Lin^-^ common myeloid progenitor cells (CMP), Lin^-^Sca-1^-^c-Kit^+^CD16/32^-^ cells, cells positive for PD-1 surface expression were calculated based on FMO gate setting. **b)** PD-L1 expression on Lin^-^ CMP cells. **c)** PD-L1 expression on Gr-1+/CD11b- cells. **d)** Percent of CD8+ T cells in S100A9Tg mice compared to WT control mice.

**Supplementary Figure S5.** **PD-1 and PD-L1 gating strategy on representative normal BM-MNCs treated with rhS100A9. a)** PD-L1 expression on erythroid precursors CD14^+^CD33^+^ cells: cells positive for PD-L1 surface expression were calculated based on FMO gate setting. **b)** PD-1 expression on progenitors: Positive PD-1 surface expression on CD34^+^ progenitors was determined using FMO gates. **c)** PD-1 expression on CD71^+^ cells. **d)** PD-1 expression on erythroid precursors CD34^+^ cells. **e)** PD-1 expression on CD71^+^ progenitors.

**Supplementary Figure S6.** **Anti-PD-L1 treatment improves hematopoiesis of aged S100A9Tg mice.** Colony formation assay was performed described in Methods and Materials. Briefly, wells of 24-well plate were coated with 5 µ g/ml anti-PD-L1 purified protein overnight at 4°C, washed three time with PBS, then 1.5 million cells of BM-MNCs in 2 ml complete media per well were cultured for 48h before colony formation assay.

**Supplementary Figure S7. Validation of Myc role in S100A9-induced PD-1/PD-L1 expression.** **a)** BM-MNCs were treated with 10 µ g/ml of IgG or anti-PD-1 BM-MNCs for 48h, CD34^+^ cells were isolated using EasySep™ Human CD34 Positive Selection Kit (Stemcell Technologies) following the manufacturer’s protocol. RNA-seq was processed as described in the Methods and Materials section. **b)** Whole cell lysis of BM-MNCs of wild type or Myc^+/-^ mice was subjected to western blotting as described in the Methods and Materials section. **c and d)**. Flow cytometry was done in supplemental figure 3.

| **Supplementary Table 1: Patient specimen characteristics** | | | |
| --- | --- | --- | --- |
| **Sex** | **Age** | **Disease Risk:** | **Karyotype:** |
| M | 77 | Intermediate | 44,XX,del(5)(q13q33),-7,-9,-12,der(15)t(12;15)(p11.2;q13),+mar[cp8]/46,XX,-9,+mar[2] |
| F | 67 | High | 44,XX,del(5)(q13q33),-7,-9,-12,der(15)t(12;15)(p11.2;q13),+mar[cp8]/46,XX,-9,+mar[2] |
| M | 74 | Good | 45,X,-Y[15]/46,XY[5] |
| M | 67 | Intermediate | //46,XX[20] |
| M | 62 | High | 46,XY[20] |
| M | 71 | High | 45,XY,add(1)(q42),add(3)(q21),der(4)t(4;12)(q22;p11.2),add(5)(q13),-6,add(7)(q10),der(12)del(12).(p11.2)t(4;12)(q22;p11.2),-14,-20,+2mar[2] |
| M | 84 | Low | 46,XY[20] |
| M | 60 | Clinical note did not specify | 46,XY[20] |
| F | 67 | Note did not specify "therapy related" per note | 46,XX,del(5)(q13q33)[7]/46,idem,der(13)add(13)(p11.2)del(13)(q12q14),add(15)(p11.2),-18,.del(20)(q11.2q13.2),?del(21)(q22.1),+22[cp9]/.46,idem,del(3)(q21),add(14)(q24),add(16)(q21),.-18,+mar[cp4] |
| M | 74 | Intermediate | 44,XY,add(3)(p11.2),del(5)(q31q35),-6,del(13)(q12q22),-20[1] /46,XY[19] |
| M | 57 | Intermediate | 46,XY,del(5)(q13q33)[2]/46,XY[18] |
| F | 63 | Intermediate | 46,XX,del(20)(q11.2)[11]/46,XX[9] |
| F | 81 | Intermediate | 47,XX,+8[20] |
| F | 79 | Low | 47,XX,+21[3]/48,XX,add(21)(p11.2)x2[2]/46,XX[27] |
| F | 71 | Low | 47,XX,+8[8]/46,XX[12] |
| M | 66 | Low | //46,XY,var(15)(p13)[20] |
| M | 84 | Low | 46,XY,del(20)(q11.2)[19]/46,XY[1] |
| M | 85 | Low | 46,XY[20] |
| F | 68 | Low | 46,XX[20] |
| F | 71 | Intermediate | //45,X,-Y[4]/46,XY[16] |
| M | 86 | High | 46,XY,del(13)(q12q22)[3]/46,XY[19] |
| M | 86 | Low | 46,X,der(Y)t(Y;1)(q12;q21)[1]/46,XY,der(7)t(1;7)(q21;q12)[1] /46,XY,der(13)t(1;13)(q21;p11.2)[1]/46,XY[17] |
| M | 54 | Intermediate | //46,XX[20] |
